# Microsecretory adenocarcinoma of the hard palate: a case report and literature review

**DOI:** 10.1186/s13000-024-01514-0

**Published:** 2024-07-09

**Authors:** Yin Lu, Yanlin Wen, Sha Feng, Wenting Huang

**Affiliations:** National Cancer Center/National Clinical Research Center for Cancer/Cancer Hospital & Shenzhen Hospital, Chinese Academy of Medical Sciences and Peking Union Medical College, Shenzhen, 518116 China

**Keywords:** Microsecretory adenocarcinoma, Salivary gland tumours, *MEF2C::SS18*

## Abstract

Microsecretory adenocarcinoma (MSA) is a new type of salivary gland neoplasm identified in the 2022 World Health Organization Classification of Head and Neck Tumour (Skalova et al., Head Neck Pathol 16:40-53, 2022) and is characterized by a unique set of histomorphologic and immunohistochemical features and a recurrent *MEF2C::SS18* fusion. MSA was initially misdiagnosed as another salivary gland tumour due to its similar morphology; until recently, only fewer than 50 cases were reported. We present a case of MSA of the hard palate with diverse architectural growth patterns, bland cytological features, abundant basophilic intraluminal secretions and fibromyxoid stroma. The tumour cells were positive for the SOX10, S100, and p63 protein and negative for the p40 protein according to immunohistochemistry. *SS18* gene rearrangement was demonstrated via break-apart fluorescence in situ hybridization. We also provided a comprehensive literature review and integrated the clinicopathological features, immunophenotype, and molecular alterations of the disease. A comprehensive understanding of MSA enables us to accurately distinguish and categorize MSA from other salivary gland tumours with analogous morphologies.

## Introduction

The widespread use of molecular assays has gradually changed the previous classification of salivary gland tumours. An increasing number of salivary gland carcinomas harbour tumour-specific gene fusions. Genetic alterations serve as the diagnostic "gold standard", enabling pathologists to redefine and accurately identify tumours that were previously misclassified, such as secretory carcinoma harbouring the *ETV6* fusion [1]. The application of molecular analysis in identifying salivary gland tumours not only enhances the tissue spectrum characteristics of tumours but also facilitates the identification of novel tumour types. The *SS18* gene encodes the SSXT protein, which is a component of the SWI/SNF chromatin remodelling complex that includes the *SS18* subunit of the BAF chromatin remodelling complex. The *SS18* break-apart FISH assay is widely available and frequently utilized as an adjunct for the diagnostic evaluation of synovial sarcoma [2, 3]. MSA consistently harbours recurrent *MEF2C::SS18* fusions, which have not been previously reported in any human neoplasm. MSA is a novel tumour entity with characteristic morphological and molecular changes. Here, we report a case of MSA of the hard palate. The tumour showed characteristic histopathological and immunohistochemical findings along with the *SS18* gene rearrangement. Previous studies have shown that MSA can be regarded as adenocarcinoma NOS, and we summarized the clinicopathological features, immunophenotype and molecular genetics of MSA through a literature review.

## Case presentation

**Fig. 1:**
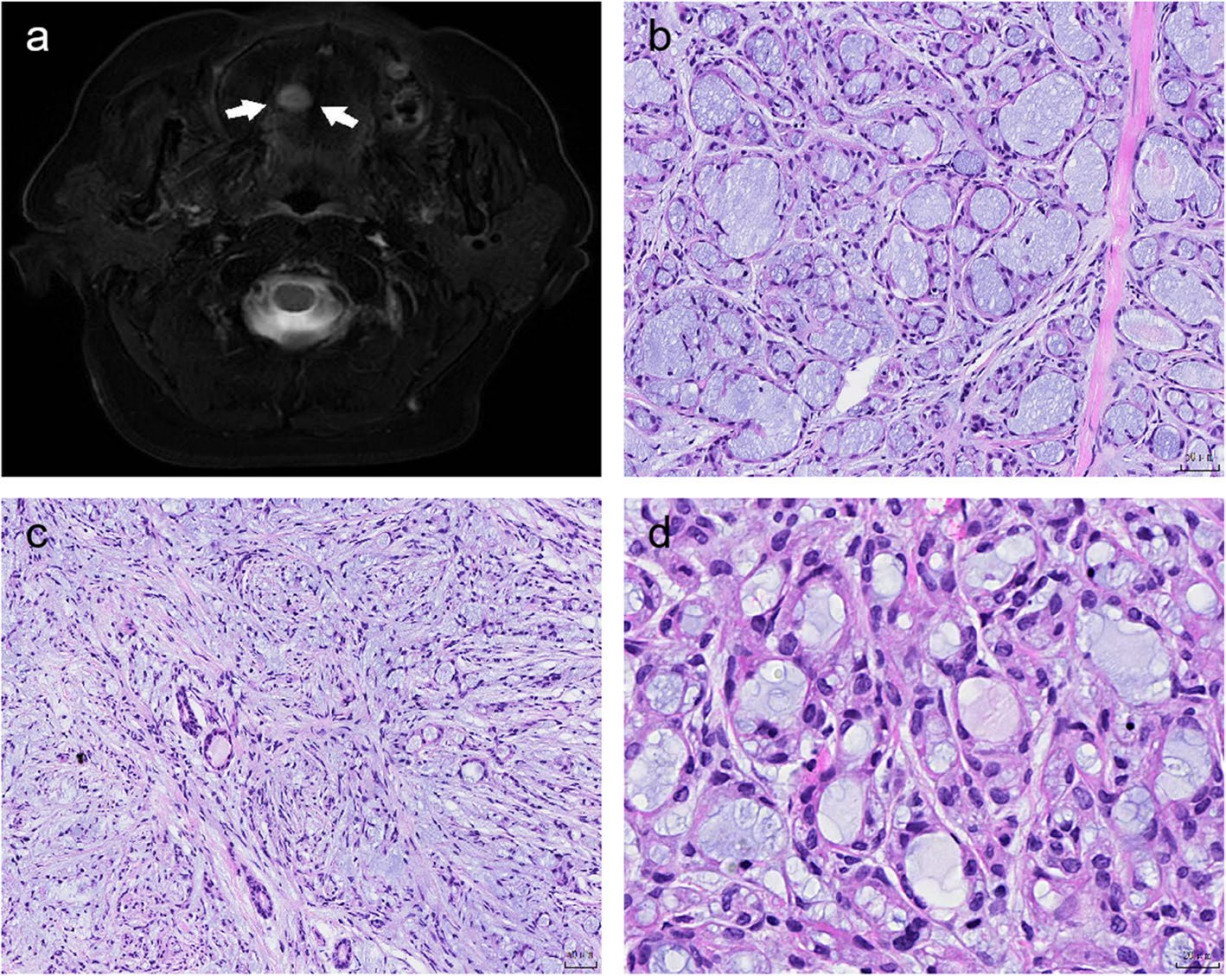
Imaging and histological morphology of MSA. MRI demonstrated a round nodule on the right side of the hard palate (**a**). The nodule was regular and well-circumscribed, with a size of about 1.1 × 1.0 × 0.9 cm. Histologically, the tumour consisted of microcysts, tubules (**b**) and cord architecture (**c**), lined with intercalated duct-like cells (**d**), which were immersed within an abundant fibromyxoid stroma

The patient, a 57-year-old woman with an unremarkable medical history, inadvertently came into contact with a mass on her hard palate three days prior. The tumour presented as a well‐circumscribed submucosal mass measuring 14 mm in diameter. An enhanced MRI revealed the presence of a round nodule on the right side of the hard palate, with dimensions of approximately 1.1 × 1.0 × 0.9 cm (Fig. 1a). The clinical differential diagnoses included pleomorphic adenoma and schwannoma. Histologically, a 14 mm diameter submucosal mass was observed within the irregular mucosal tissue. The sections appeared white and solid. The tumour exhibited an unencapsulated and well-circumscribed appearance at low-power magnification; however, upon closer examination, focal permeative infiltration at the periphery of the tumour and involvement of the adjacent salivary gland tissue were revealed. Perineural invasion was observed when there was no evidence of lymphovascular invasion. The tumour exhibited a complex architecture consisting of microcysts, tubules (Fig. 1b) and cord (Fig. 1c) patterns, all lined with intercalated duct-like cells (Fig. 1d). The tumour cells exhibited eosinophilic or clear cytoplasm and oval nuclei with minimal atypia and were immersed within an abundant fibromyxoid stroma. Basophilic secretions were observed in the lumen. The mitotic rates were markedly low, and necrosis was absent.

**Fig. 2:**
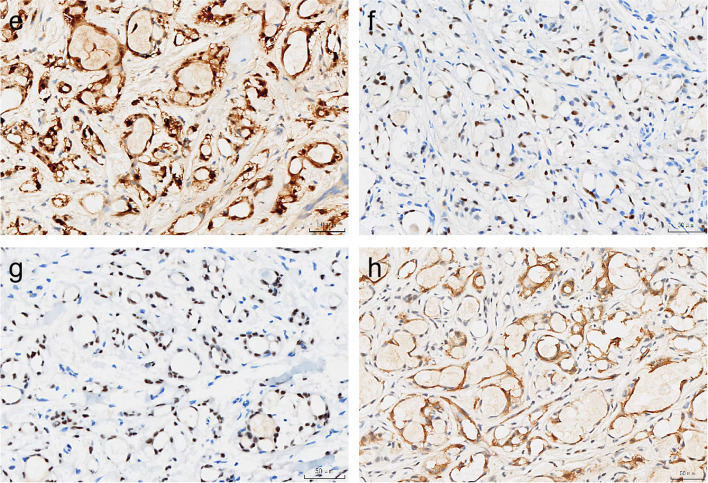
Immunohistochemistry of MSA. The tumour cells were strongly positive for S-100 (**e**), p63 (**f**), SOX-10 (**g**) and focal SMA (**h**) protein by immunohistochemistry

**Fig. 3:**
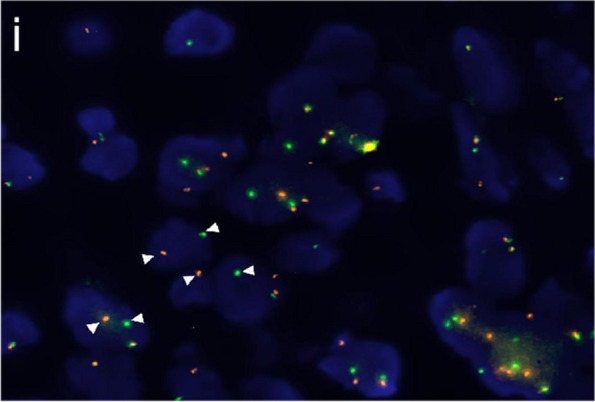
*SS18* break-apart FISH of MSA. FISH analysis demonstrated positive *SS18* break-apart signals (arrowheads pointing at red and green split-apart signals) (i)

Immunohistochemically, the tumour cells were diffusely positive for CK7, S-100 (Fig. 2e), p63 (Fig. 2f), SOX10 (Fig. 2g) protein and focally positive for SMA (Fig. 2h) protein and completely negative for CK20, p40, Mammaglobin, CD117 and GCDFP15. The *SS18* break-apart FISH assay was performed on this patient, revealing a distinct pattern of separation indicative of gene rearrangement (Fig. 3i).

The postoperative course proceeded without complications, and there was no evidence of recurrence or metastasis at the 20-month follow-up.

## Review of the literature

To the best of our knowledge, 42 cases of MSA have been previously reported, and they are summarized in Table 1. Including our patient, the median age was 53 years (ranging from 17 to 89 years), with similar proportions of males and females. MSA primarily arose in the oral cavity (81%, 35/43) and included the buccal, palate, and retromolar trigones as well as the angle of the mandible and parotid gland. However, the occurrence of 8 cases was observed in distinct extraoral sites. The tumour sizes ranged from 0.5 cm to 5.3 cm (median size: 0.98 cm). Most patients underwent surgical resection. The shared histologic features included intercalated duct-like cells exhibiting eosinophilic to clear cytoplasm; small, uniform oval nuclei; microcysts; tubules; cribriform and cord growth patterns; abundant intraluminal secretions; and fibromyxoid stroma.

**Table 1:** Clinical features of microsecretory adenocarcinoma

Case	N	Sex	Age(years)	Tumour location	Tumour size(cm)	Follow-up(months)
Bishop, J.A. et al. [4]	*n* = 5	M:2 F:3	21–80	Buccal Palate Parotid gland	0.8–2.2	N.A
Kawakami, F. et.al. [5]	*n* = 1	F	37	Palate	1.5	NED (4)
Bishop, J.A. et al. [6]	*n* = 24	M:11 F:13	17–83	Buccal Palate Retromolar trigone Parotid gland Angle of mandible	0.6–3.0	NED (1–216)
Dibbern, M.E. et.al [7]	*n* = 1	F	89	External auditory canal	N.A	N.A
Jurmeister, P., et al. [8]	*n* = 1	M	61	Buccal	N.A	NED (144)
He XJ, et al. [9]	*n* = 1	M	27	Buccal	2	NED (11)
Gui, H., et al. [10]	*n* = 1	M	70	Palate	5.3	NED (20)
Bishop, J.A., et al. [11]	*n* = 4	M:4	61–74	Nose Chin Posterior scalp External auditory canal	0.5	NED (4–6)
An FX, et al. [12]	*n* = 1	M	48	Palate	1.5	N.A
Bogiatzi, S. et al. [13]	*n* = 3	M:1 F:2	53–78	Forearm Fourth finger of the hand Vertex	0.9–1.3	NED (12–180)
Present	*n* = 1	F	57	Palate	1.4	NED (20)
Total	*n* = 43	M: F = 22:21	17–89(median:53)	Palate(*n* = 20) Buccal(*n* = 10) Retromolar trigone(*n* = 2) Parotid gland(*n* = 2) …	0.5–5.3(median:0.98)	NED (1–216) (median:12)

*F* Female, *M* Male, *N.A.* not applicable, *NED* no evidence of disease

Immunohistochemical and molecular findings are shown in Table 2. The tumours exhibited a remarkably consistent immunohistochemical profile. Tumour cells were positive for S-100 (100%, 41/41, 36 for diffuse positive and 5 for focal positive), diffuse SOX10 (100%, 23/23), p63 (100%, 43/43, 33 for diffuse positive and 10 for focal positive) and variable SMA (42%, 10/24) protein, whereas only a few patients were positive for p40 protein. Table 3 illustrated the findings, wherein 96% (22/23) patients exhibited *SS18* gene rearrangement via in situ hybridization. And *MEF2C::SS18* fusion was identified in all patients (100%, 35/35). A significant group (88%) shared identical break points, occurring at exon 7 of *MEF2C* and exon 4 of *SS18*. However, a subset of patients displayed breakpoints at alternative locations: exon 7 of *MEF2C* and exon 2 of *SS18*, exon 5 of *MEF2C* and exon 4 of *SS18*, as well as exon 10 of *MEF2C* and exon 5 of *SS18*. There were no cases of disease-induced death among the patients who were followed up. The median follow-up time was 12 months (ranging from 1 to 216 months), and only one patient [8] was reported to have local recurrence and pulmonary metastases.

**Table 2:** Immunohistochemical findings of microsecretory adenocarcinoma

Case	N	S-100	SOX10	p63	p40	SMA
Bishop, J.A., et al. [4]	*n* = 5	100% (5/5)	N.A	100% (5/5)	0% (0/5)	0% (0/5)
Kawakami, F., et al. [5]	*n* = 1	100% (1/1)	100% (1/1)	100% (1/1)	0% (0/1)	0% (0/1)
Bishop, J.A., et al. [6]	*n* = 24	100% (24/24)	100% (14/14)	100% (24/24)	0% (0/21)	21% (4/19)
Dibbern, M.E., et al. [7]	*n* = 1	100% (1/1)	N.A	100% (1/1)	0% (0/1)	N.A
Jurmeister, P., et al. [8]	*n* = 1	N.A	100% (1/1)	100% (1/1)	N.A	N.A
He XJ, et al. [9]	*n* = 1	100% (1/1)	100% (1/1)	100% (1/1)	0% (0/1)	0% (0/1)
Gui, H., et al. [10]	*n* = 1	100% (1/1)	100% (1/1)	100% (1/1)	0% (0/1)	0% (0/1)
Bishop, J.A., et al. [11]	*n* = 4	100% (4/4)	N.A	100% (4/4)	100% (2/2)	100% (2/2)
An FX, et al. [12]	*n* = 1	100% (1/1)	100% (1/1)	100% (1/1)	0% (0/1)	100% (1/1)
Bogiatzi, S., et al. [13]	*n* = 3	100% (2/2)	100% (3/3)	100% (3/3)	50% (1/2)	67% (2/3)
Present	*n* = 1	100% (1/1)	100% (1/1)	100% (1/1)	N.A	100% (1/1)
Total	*n* = 43	100% (41/41)	100% (23/23)	100% (43/43)	3% (1/35)	42% (10/24)

**Table 3:** Molecular findings of microsecretory adenocarcinoma

Case	n	FISH(*SS18*)	RNA-Seq/PCR	Fusion Break-points
Bishop, J.A., et al. [4]	*n* = 5	N.A	100% (5/5)	exon 7 of the *MEF2C* gene and exon 4 of the *SS18* gene (*n* = 1)
Kawakami, F., et al. [5]	*n* = 1	N.A	100% (1/1)	exon 7 of the *MEF2C* gene and exon 4 of the *SS18* gene (*n* = 1)
Bishop, J.A., et al. [6]	*n* = 24	93% (13/14)	100% (21/21)	exon 7 of the *MEF2C* gene and exon 4 of the *SS18* gene (*n* = 24)
Dibbern, M.E., et al. [7]	*n* = 1	100% (1/1)	N.A	N.A
Jurmeister, P., et al. [8]	*n* = 1	100% (1/1)	100% (1/1)	N.A
He XJ, et al. [9]	*n* = 1	100% (1/1)	N.A	N.A
Gui, H., et al. [10]	*n* = 1	100% (1/1)	100% (1/1)	exon 5 of the *MEF2C* gene and exon 4 of the *SS18* gene (*n* = 1)
Bishop, J.A., et al. [11]	*n* = 4	100% (3/3)	100% (3/3)	exon 7 of the *MEF2C* gene and exon 4 of the *SS18* gene (*n* = 2) exon 10 of the *MEF2C* gene and exon 5 of the *SS18* gene (*n* = 1)
An FX, et al. [12]	*n* = 1	100% (1/1)	N.A	N.A
Bogiatzi, S., et al. [13]	*n* = 3	N.A	100% (3/3)	exon 7 of the *MEF2C* gene and exon 2 of the *SS18* gene (*n* = 2) exon 7 of the *MEF2C* gene and exon 4 of the *SS18* gene (*n* = 1)
Present	*n* = 1	100% (1/1)	N.A	N.A
Total	*n* = 43	96% (22/23)	100% (35/35)	exon 7 of the *MEF2C* gene and exon 4 of the *SS18* gene (*n* = 29, 88%) exon 7 of the *MEF2C* gene and exon 2 of the *SS18* gene (*n* = 2, 6%) exon 5 of the *MEF2C* gene and exon 4 of the *SS18* gene (*n* = 1, 3%) exon 10 of the *MEF2C* gene and exon 5 of the *SS18* gene (*n* = 1, 3%)

## Discussion

The MSA was first proposed by Bishop et al. [4] in 2019 and was formally included as a novel entity of salivary gland tumours in the most recent World Health Organization Classification of Head and Neck Tumours. MSA usually occurs in the oral cavity, especially in the minor salivary glands; however, identifying MSA in an extraoral location is not rare. The primary tumour location was in the skin, which expands the sites that can be affected by MSA, including the nose, chin, posterior scalp [11], external auditory canal [7, 11], forearm, fourth finger of the hand and vertex [13]. These cutaneous tumours are identical to those described in the salivary glands, with unique histopathologic features and molecular underpinnings. Bogiatzi et al. [13] suggested that MSA of the skin (MSAS) is a skin homologue of the salivary gland. The MSA is composed of tubules, microcysts and cord structures with monotonous bland cells and sometimes presents with focal infiltration and perineural invasion. Many studies suggest that no reported cases have shown recurrence or metastasis after surgical resection [6], although prognostic information is limited. However, some cases [10, 11] demonstrate high-grade morphology as a solid growth pattern, significant nuclear atypia, an elevated mitotic rate, and necrosis. Gui, H., et al. [10] presented a neoplasm with aggressive biological behaviour in the left maxillary region. There was another similar case [8] in which local recurrence and pulmonary metastases occurred. However, the minor progress during long-term follow-up of this patient strongly confirms that MSA is a low-grade salivary gland tumour.

In our patient, we observed that tumour cells expressed SOX10 and S-100 protein, which confirmed that the tumour was intercalated duct differentiation. Unexpectedly, the tumour cells focally expressed SMA protein, which is a specific marker of myoepithelial differentiation. Some reports in the literature have also demonstrated similar expression of SMA protein [6, 12]. Bishop et al. [6] presented that this finding does not indicate true myoepithelial differentiation, but rather exhibits occasional nonspecific reactivity in intercalated duct-like tumours. Interestingly, Bishop et al. [11, 14] indicated that a subgroup of cutaneous MSA displayed a genuine myoepithelial layer. The intercalated duct is the terminal branch of the salivary gland. There is a heterogeneous immunophenotype (p63 + /p40-) observed in MSA. This phenomenon can be explained by two factors. First, p63 is usually expressed in the basal layer of stratified epithelium, myoepithelial cells, and neoplasms that derive from these epithelia [15]. However, it is not a rare event to observe the expression of p63 in both true and pseudo-myoepithelial cells. Additionally, p40 antibody, an isotype of p63, exhibits a higher specificity for basal and true myoepithelial cells [16]. Therefore, the heterogeneous expression (p63 + /p40-) indicates a lack of myoepithelial or basal differentiation in the tumour. Second, the heterogeneous immunophenotype (p63 + /p40-) may represent a specific state of tumour progenitor cells, similar to the amplifying progenitor cells of intercalated duct cells. Several studies [17–19] have reported that similar immunoprofile (p63 + /p40-) was also observed in polymorphous adenocarcinoma (PAC). Notably, the p63/p40 staining patterns are not consistent. Different from MSA, the p63 positivity of PAC is quite variable, and the p63 staining pattern is not biphasic.

MSA was previously grouped as “adenocarcinoma, not otherwise specified” (NOS) and classified as a unique pathologic entity by recognition of recurrent *MEF2C::SS18* fusion. Within the *SS18* gene fusion family, microcribriform adenocarcinoma (MCA) was recently proposed entity by Weinreb et al. [20], shared morphological and molecular features with microsecretory adenocarcinoma (MSA). There are similar features in both tumours, such as infiltrative characteristics at the edge, a cellular fibromyxoid stroma, bland cellular features, basophilic intraluminal secretions, and various architectural growth patterns. However, there are also differences between them. Microcysts and tubule structures are more prominent in MSA, with more abundant intraluminal secretions, while solid, cribriform, small clusters, and single cell patterns were often observed in MCA. Indeed, rare structural features such as cribriform structures can also be observed in MSA [4], similar to those found in MCA. Immunohistochemically, p63 protein is usually positive in MSA but usually negative in MCA, though focal p63 expression was observed in a case of MCA with *SS18::ZBTB7A*, suggesting focal biphasic growth [20]. At the molecular level, there are differences in *SS18* fusion partners (*MEF2C::SS18* for MSA and *SS18::ZBTB7A* for MCA) and gene position (3' for MCA and 5' for MSA). Exon 4 is predominantly manifested in MSA while exon 10 is primarily observed in MCA. Additionally, the differential diagnosis of MSA should include various salivary gland tumours exhibiting secretory features and microcystic and tubular structures. As a novel entity described in the new classification, sclerosing microcystic adenocarcinoma (SMA) [21, 22] bears some resemblance to MSA in terms of its microcystic and cord-like growth patterns. However, SMA is a biphasic tumour with both ductal and myoepithelial cells. Sclerotic or fibrous stroma is also a distinct feature. However, the genetic mechanisms of SMA pathogenesis remain to be elucidated. Similar to MSA, secretory carcinoma can also exhibit microcystic and tubular structures. Secretory carcinoma, however, exhibited a uniformly eosinophilic appearance, whereas the secretion of MSA displayed a significantly basophilic character. Unlike in MSA, secretory carcinomas usually express the Mammaglobin and NTRK protein according to immunohistochemistry and frequently exhibit a distinct molecular alteration known as the *ETV6::NTRK3*, *ETV6::RET* or *ETV6::MET* fusion gene [23–28]. The cribriform subtype of polymorphous adenocarcinoma (PAC), which exhibits a microcystic architecture resembling MSA, demonstrates alterations in *PRKD1, 2, or 3* [21, 29–31]. Different from MSA, PAC is characterized by single columns, solid, cribriform architectures. Papillary structures and peripheral palisading may be observed. Finally, adenoid cystic carcinoma, a biphasic cell tumour with frequent rearrangement of the *MYB* or *MYBL1* gene [32–34], is an important differential diagnosis of MSA. *SS18* break-apart fluorescence in situ hybridization (FISH) is frequently used as an adjunct for diagnosing synovial sarcoma. A comprehensive study [35] indicated that while FISH was performed on 4 MSA and 8 tissue microarrays (TMAs) containing 423 salivary gland carcinomas, all 4 MSA demonstrated classic split patterns on *SS18* break-apart FISH, and other tumours were negative for *SS18* rearrangement. Therefore, the author proposed that the available *SS18* break-apart FISH assays can be used to diagnose MSA in a highly sensitive and specific manner, which would obviate the need for NGS techniques. In this study, the break-apart signal detected by FISH confirmed the association of the tumour with the *SS18* gene. Combined with the location, morphology and immunophenotype of the tumour, we concluded a diagnosis of MSA. Unfortunately, due to limited laboratory resources, we were unable to conduct RNA-seq/PCR testing for further analysis in these patients. Moreover, in order to explore the molecular alterations in MSA in this review, we compiled and analyzed data from previously reported patients (presented in Table 3). Through RNA-Seq/PCR, *MEF2C::SS18* was detected in all the patients, underscoring its cost-effectiveness, high sensitivity and specificity. Notably, exon 7 of the *MEF2C* gene and exon 4 of the *SS18* gene emerged as the most common fusion breakpoints, a finding that surprised us.

The present article aims to improve the understanding of MSA disease in terms of morphology, immunophenotype, and molecular genetics, facilitating accurate identification of salivary gland tumours.

## Data Availability

No datasets were generated or analysed during the current study.
